# Toughening Behavior Investigation of Fish Scale-Inspired Composite Structure with Overlapping Helical Architecture

**DOI:** 10.3390/biomimetics11090633

**Published:** 2026-09-04

**Authors:** Zhiquan Wei, Xinlan Hu, Xinran Hu, Yaozhe Yu

**Affiliations:** 1 School of Aeronautics and Astronautics, Tiangong University, Tianjin 300387, China; xin_lann@163.com (X.H.); 18836940010@163.com (Y.Y.); 2 Tianjin Area Major Laboratory of Advanced Mechatronics Equipment Technology, Tianjin 300387, China; 3 School of Mechanical Engineering, Tiangong University, Tianjin 300387, China; 15833244394@163.com

**Keywords:** bio-inspired design, architected composite, fish scales, 3D printing, fracture behavior, toughening mechanisms

## Abstract

The inherent trade-off between strength and toughness in structural materials remains a critical challenge. Inspired by the hierarchical architecture of fish scales, this study proposes a novel overlapping helical composite structure. Multi-material three dimensional (3D) printing technology was employed to fabricate single-edge notched bending specimens. Quasi-static three-point bending experiment was conducted to investigate the mechanical performance of a fish scale-inspired structure. The results show that compared to the stiff bulk structure, the bio-inspired design exhibits a 60.4% enhancement in apparent fracture toughness and a 157.5% increase in energy absorption despite a reduction in flexural modulus and strength. The significant improvement may be attributed to the synergistic effects of crack deflection, which transform the fracture mode from catastrophic brittle failure to progressive damage with a stable post-peak deformation stage. Furthermore, parametric studies reveal that both the linear helical angle and its nonlinear gradient distribution critically govern the toughening efficiency. An optimal linear angle of 19° provides the best overall performance, while a nonlinear gradient (*e* = 1.75) further shifts energy dissipation towards the post-peak deformation stage, achieving a higher toughening efficiency. This work establishes a fundamental understanding of an overlapping helical coupling toughening strategy and provides a promising design route for high-damage-tolerance composite structures.

## 1. Introduction

The inherent conflict between strength and toughness in structural materials remains one of the long-standing fundamental challenges in the field of materials science [1,2]. High strength is often accompanied by low toughness and limited deformability, while tough materials typically sacrifice stiffness and strength [3]. This inverse relationship severely restricts the engineering application of advanced structural materials under complex loading conditions. However, conventional homogeneous materials struggle to reconcile these two conflicting performance requirements. Some biological materials in nature provide effective strategies for addressing this challenge [4,5]. Through long-term evolution, biological structures such as shells, bone, and fish scales achieve the synergistic optimization of strength and toughness by precisely assembling brittle mineral phases with ductile organic phases, with overall mechanical performance far exceeding the simple superposition of individual components.

Among numerous biological designs, the mud-brick architecture in nacre is one of the earliest and most extensively studied configurations. Composed of brittle aragonite tablets alternately stacked with only 5% soft organic matrix, nacre achieves a fracture toughness more than three orders of magnitude higher than that of pure aragonite [6,7]. Gu et al. [8] fabricated this kind of mud-brick composite structure via 3D printing. A drop-weight impact experiment and simulation demonstrated that homogeneous stiff materials undergo brittle fracture due to stress concentration, whereas the mud-brick structure substantially enhanced the energy dissipation through crack deflection and interfacial delamination, achieving a 30% increase in energy absorption. Chan-Colli et al. [9] pointed out that the mud-brick plate improved the ballistic performance by producing a more ductile failure and enabling localized energy absorption via the plastic deformation of tablets and globalized energy dissipation due to interface debonding and friction. In addition, the conch shell possesses a hierarchical cross-lamellar pattern that endows it with a fracture toughness two to three orders of magnitude greater than that of its mineral phase [10]. Zou et al. [11] found that the tensile strength and fracture toughness of the cross-lamellar architecture were 24% and 89% higher than its stiff constituent, respectively. The Bouligand (helical) structure, inspired by the mantis shrimp dactyl club, has also attracted considerable attention [12,13,14]. Wu et al. [15] designed and fabricated notched helical structures via 3D printing. Three-point bending experiments revealed that the Bouligand structure induces a mixed mode I+II+III fracture locally, leading to significantly enhanced fracture toughness. This structure promotes energy dissipation by inducing crack twisting, which increases the crack propagation area [16,17,18]. Beyond these aforementioned architecture, other notable configurations include the beetle elytra-inspired interlocking structure [19], the sponge-inspired layered structure [20], the turtle shell or cuttlebone-inspired suture structure [21,22], and the bone-inspired concentric hexagonal structure [23]. Each configuration exhibits distinct toughening mechanisms, providing rich design space for tailoring the performance of soft–stiff composite structures.

Although bio-inspired composite structures have been widely proposed and optimized, demonstrating excellent mechanical performance, most previous studies have focused on a single feature of a single biological material. Since different structures possess distinct energy dissipation mechanisms, imitating only a single biological feature in a soft–stiff composite design still limits further performance improvement. In recent years, some researchers have attempted to fuse the toughening characteristics from different configurations, developing hybrid designs that outperform single bio-inspired architecture. Jia et al. [23] combined layered and concentric hexagonal structures, finding that the hybrid configuration achieves a 49% increase in energy absorption over the single mud-brick architecture. Zhang et al. [24,25] hybridized the hierarchical cross-lamellar structure with the Bouligand structure, achieving the synergistic enhancement of strength and toughness by more than 500%. Moreover, the hybrid design may also involve the fusion of design strategies and structures, such as a gradient design with a mud-brick structure [26,27], a gradient design with an interlocking structure [19], and a rigidity–toughness coupling design with a mud-brick structure [28]. Inspired by the gradient variation of mechanical properties in the mantis shrimp dactyl club, we also designed a hybrid structure combining a bulk top layer, a mud-brick middle layer, and a layered bottom layer, achieving rigidity–toughness coupling with a 375% increase in energy absorption over the mud-brick structure [29]. These studies demonstrate that the synergistic effects among different toughening mechanisms can overcome the performance limits of a single configuration. However, the effectiveness of hybrid designs hinges on a thorough understanding of the distinct toughening mechanisms across different architectures, and existing hybridization attempts are largely empirical combinations.

Fish scales, as a typical biological protective structure, offer new insights for overcoming the above limitations. Unlike nacre or conch shells, fish scales achieve a balance between protective performance and flexibility [30]. Under loading, adjacent scales come into contact, rotate, and eventually enter an interlocked state. This process not only promotes stress transfer and redistribution but also dissipates substantial energy through interfacial sliding between scales [31,32]. Studies have shown that the puncture resistance of a fish scale-inspired overlapping structure can be enhanced dozens of times compared with a homogeneous structure, with extremely limited loss of flexibility [33,34,35,36]. Furthermore, the microstructure of fish scales commonly incorporates helical arrangements, in which collagen fiber layers rotate successively along the thickness direction to form a helical structure [37]. This dual structural characteristic, namely macroscopic overlapping arrangement and microscopic helical stacking, endows fish scales with unique advantages in resisting crack penetration. However, existing studies on fish scale-inspired structures have primarily focused on the effects of parameters (e.g., scale inclination angle and overlap ratio) on macroscopic stiffness and puncture resistance [38,39,40], with limited systematic investigation on the synergistic effects between outer overlapping and inner helical architectures.

In this paper, inspired by the hierarchical architecture of fish scales, a novel soft–stiff composite structure featuring an overlapping helical configuration is proposed. This design uniquely integrates the overlapping architecture with helical arrangement. Multi-material 3D printing technology was employed to fabricate the intricate fish scale-inspired structures with different configurations. A quasi-static three-point bending experiment was conducted to investigate the mechanical performance of a fish scale-inspired structure. By comparing the mechanical response and high-resolution fracture morphology of the fish scale-inspired structure with that of the stiff bulk structure, the underlying toughening mechanisms of the fish scale-inspired structure are inferred based on the experimental observations. Furthermore, parametric studies are performed to evaluate the effects of both linear and nonlinear helical angles on the overall performance. This work seeks to establish a fundamental understanding of the overlapping helical toughening strategy and to provide a crucial reference for the design of high-damage-tolerance bio-inspired composite structures.

## 2. Materials and Methods

### 2.1. Geometric Models of Fish Scale-Inspired Structure

**Figure 1:**
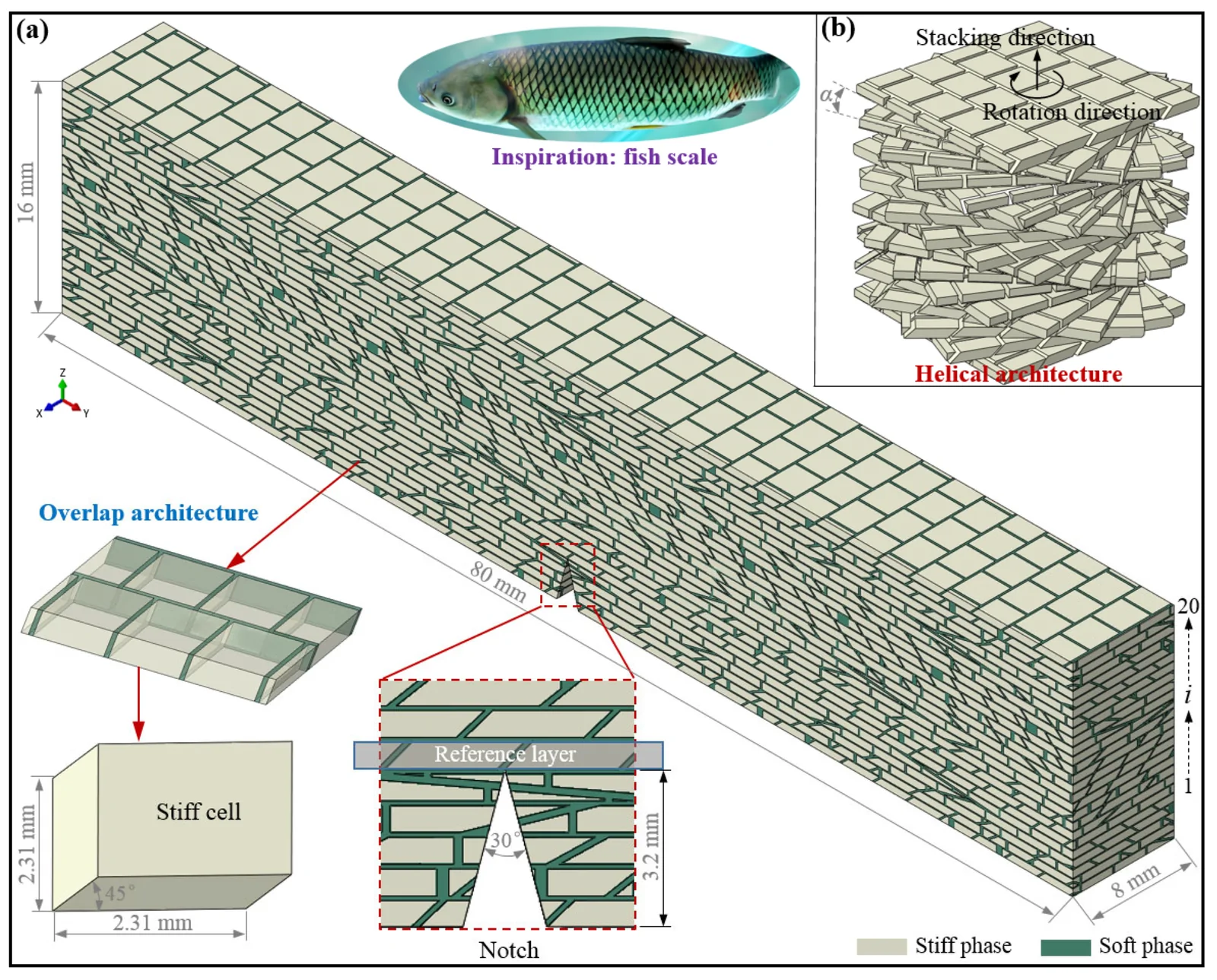
Fish scale-inspired composite structure. (**a**) SENB configuration. (**b**) Schematic diagram of helical architecture.

Following the ASTM E1820 standard, the single-edge notched bending (SENB) configuration was adopted in this study. The overall dimensions are 80 mm in length, 8 mm in width, and 16 mm in thickness. An isosceles triangular notch with a height of *a*_0_ = 3.2 mm and an apex angle of 30° was prefabricated at the mid-span of the bottom surface to induce crack initiation. Figure 1 presents the schematic diagrams of the fish scale-inspired composite structure. The model consists of twenty-five soft–stiff composite layers, with twenty layers above the notch serving as the complete load-bearing region and five layers below the notch for the prefabricated notch region. Adjacent composite layers are separated by soft adhesive layers. Each composite layer has a thickness of 0.55 mm, and each adhesive layer has a thickness of 0.09 mm. Each composite layer consists of a stiff part and a soft cohesive part, with the latter serving as an intra-layer binder. In detail, the composite layer comprises multiple stiff cells separated by soft cohesive material. Each complete stiff cell is an oblique hexahedron with planar dimensions of 2.31 mm in length and 2.31 mm in width and an inclination angle of 45°. All stiff cells within a given composite layer share the same inclination direction. Adjacent cells partially overlap, which mimics the imbricated features of fish scales. Regarding the helical stacking sequence along the thickness direction, the lowest composite layer above the notch is defined as the reference layer (0°), with subsequent composite layers stacked sequentially and rotated by an angle of *α* relative to the previous composite layer. The helical angle *α_i_* of the *i*-th composite layer (*i* = 1, 2, 3, …, 20) relative to the fixed reference layer can be calculated as:

(1) $$\alpha_{i}=360^{\circ }\cdot {\left(\frac{i-1}{19}\right)}^{e},$$

where *e* is the power-law exponent, and *e* = 1.0 is the linear helical structure. A complete linear helical cycle is achieved over the 20 layers above the notch, with the rotation angles relative to the fixed reference layer following the sequence of [0°/19°/38°/…/360°]; i.e., *α* = 360°/19 ≈ 19°. For nonlinear (gradient) distributions, the power-law exponent *e* governs the variation of *α_i_* along the thickness direction, as discussed in Section 4.2. The volume fraction of soft material is strictly controlled at approximately 24% across all composite structures in this study. For comparison, a reference bulk structure with the same external dimensions and notch geometry was also designed. However, unlike the fish scale-inspired structure, the bulk structure is composed entirely of stiff material without any soft material, serving as a conventional homogeneous material baseline.

### 2.2. Sample Preparation

All specimens are fabricated using the Objet260 Connex multi-material 3D printer (Stratasys Ltd., Rehovot, Israel), which offers a layer resolution of 16 μm and an in-plane printing accuracy of 600 dpi, enabling the high-fidelity reproduction of intricate architectural details. During the printing process, VeroWhite (a brittle photopolymer, Stratasys) and Agilus30 (a hyperelastic photopolymer, Stratasys) are simultaneously jetted through separate print heads and instantly cured under ultraviolet irradiation. After printing, the specimens are carefully detached from the build platform, and the sacrificial support material is removed using running water. Subsequently, all specimens are dried at room temperature for 24 h to eliminate any residual moisture and to stabilize their mechanical response prior to testing. To ensure a fair comparison, the reference bulk specimens are fabricated using the same printing parameters and within the same batch as the fish scale-inspired specimens. After drying, the actual dimensions of all specimens are measured individually using a digital caliper with an accuracy of ± 0.01 mm. The measured dimensions are 80.00 ± 0.05 mm in length, 8.00 ± 0.02 mm in width, and 16.00 ± 0.03 mm in thickness, with no significant difference observed across different configurations, confirming the dimensional consistency of the printed specimens. In addition, all specimens are weighed using a precision electronic balance. The measured mass of each specimen is 12.26 ± 0.09 g, with no significant difference observed across different configurations, further confirming the comparability of all tested configurations.

### 2.3. Quasi-Static Three-Point Bending Experiments

**Figure 2:**
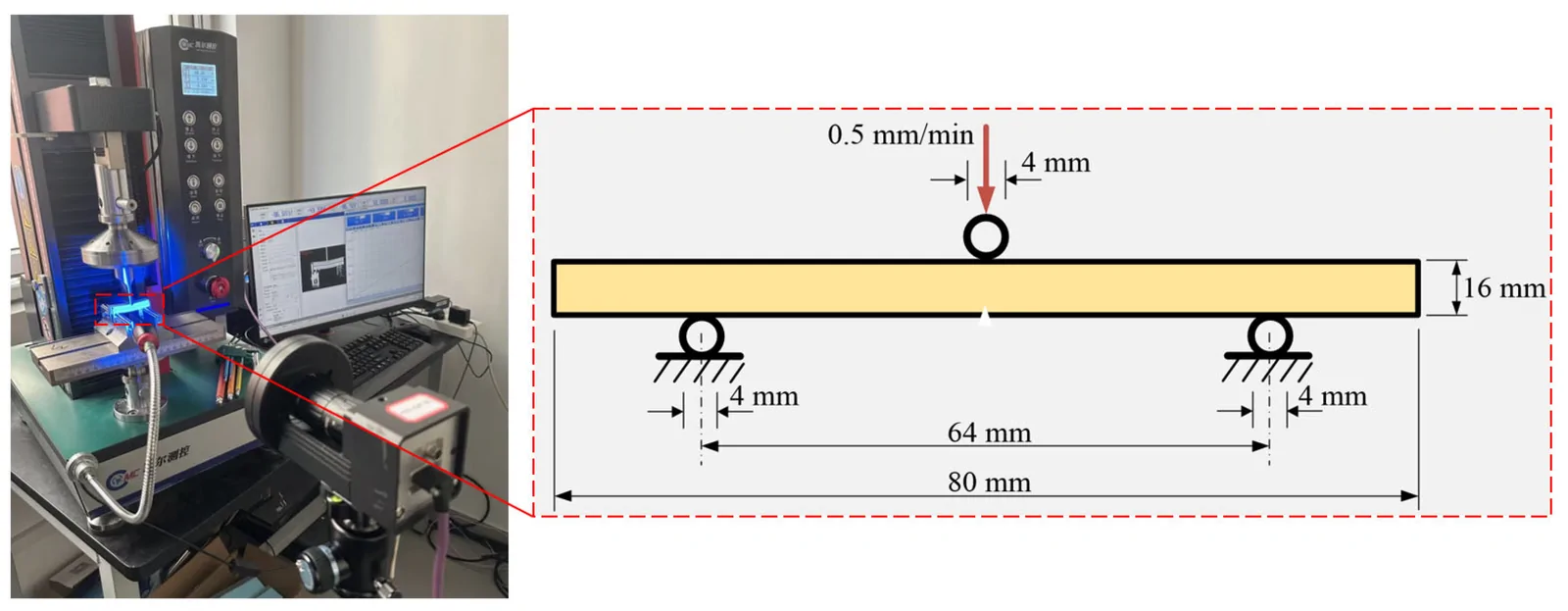
Quasi-static three-point bending test.

As shown in Figure 2, quasi-static three-point bending experiments are carried out utilizing a vertical universal electronic material testing machine (CARE MC Ltd., Tianjin, China). The distance between the two lower supporting rollers (support span) is fixed at 64 mm. Both the loading indenter and the bottom supports are equipped with cylindrical contact surfaces featuring a curvature radius of 2 mm. A displacement-controlled loading mode is adopted at a constant crosshead speed of 0.5 mm/min, ensuring that the experiments are performed under quasi-static conditions. The loading is terminated when either specimen fracture occurs or the maximum crosshead displacement reaches 8 mm, whichever comes first. During the entire bending process, the built-in data acquisition system operates at a sampling rate of 30 Hz, synchronously recording the applied force and corresponding crosshead displacement, thereby generating the complete force–displacement response for each test. In parallel, a high-resolution camera is positioned normal to the specimen’s lateral surface, capturing the deformation and cracking events at a frame rate of 12 frames per second. The acquired time-stamped image sequences enable a precise retrospective analysis of the entire fracture process, facilitating the identification of crack initiation sites, the tracking of subsequent growth paths, and the examination of resulting fracture surface morphologies. To ensure the statistical reliability and reproducibility of the measured data, five replicate specimens are tested independently for each structural configuration. All tests are conducted under stable ambient conditions (temperature of 23 ± 2 °C and relative humidity of 50 ± 5%) to minimize environmental interference with the mechanical behavior of polymeric composite materials. It should be emphasized that the constituent polymers (VeroWhite and Agilus30) are inherently sensitive to loading rate [40]. Consequently, the mechanical responses and comparative rankings reported herein are strictly representative of the quasi-static regime adopted in this study (0.5 mm/min). The applicability of the observed toughening mechanisms in the fish scale-inspired structure to higher strain rates will be addressed in future work.

### 2.4. Calculation of Performance Parameters

To characterize the quasi-static mechanical performance of the structure, six mechanical parameters are derived from the recorded force–displacement curves, including flexural modulus and flexural strength, crack initiation displacement, apparent fracture toughness, total energy absorption, and post-peak energy dissipation ratio. These indices collectively enable a quantitative assessment of stiffness, strength, deformability, crack resistance, and energy dissipation capacity. All measured data in the text are reported as mean ± standard deviation (SD). Statistical comparisons between different configurations are performed using one-way analysis of variance (ANOVA), with *p* < 0.05 considered statistically significant. The *p* value represents the probability of observing the current or more extreme results under the null hypothesis of no difference between groups; a smaller *p* value indicates stronger evidence against the null hypothesis.

The flexural modulus ($E_{f}$) quantifies the bending stiffness, representing the resistance to elastic deformation under flexural loading. It is determined from the slope of the initial linear-elastic regime of the force–displacement curve. Based on the Euler–Bernoulli beam theory, $E_{f}$ is calculated as:

(2) $$E_{f}=\frac{L^{3}k}{4BW^{3}},$$

where *L* is the support span; *B* and *W* are the specimen width and thickness, respectively; and *k* denotes the initial slope of the force–displacement curve.

The flexural strength ($\sigma_{b}$) represents the maximum bending stress sustained by the specimen prior to failure. Based on the Euler–Bernoulli beam theory, the flexural strength for a rectangular beam under three-point bending is calculated as:

(3) $$\sigma_{b}=\frac{3F_{\max}L}{2BW^{2}},$$

where $F_{max}$ is the peak force. This parameter characterizes the ultimate load-bearing capacity of each structure.

The crack initiation displacement ($\delta_{ini}$) is defined as the displacement at which the macroscopic crack first occurs. To establish a consistent and objective criterion across all specimens, the initiation point is identified as the first force drop exceeding 2% of the peak force on the force–displacement curve, and the corresponding displacement is taken as $\delta_{ini}$. This parameter reflects the ability to resist crack initiation.

The apparent fracture toughness ($K_{app}$) is employed to evaluate the fracture resistance, which characterizes the resistance to stable crack growth. It is calculated following the data reduction procedures outlined in ASTM E1820, based on the *J*-integral theory from elastic–plastic fracture mechanics:

(4) $$K_{\mathrm{app}}=\sqrt{\frac{E\cdot J}{1-\nu^{2}}},$$

where *E* and *υ* are the elastic modulus and Poisson’s ratio of the material, respectively. For the bulk structure, which is composed entirely of VeroWhite, the values of *E* = 1.2 GPa and *υ* = 0.3 are directly used. For the fish scale-inspired composite structures, which are heterogeneous and anisotropic due to the incorporation of the soft Agilus30 phase and the overlapping helical architecture, a direct application of the ASTM E1820 formulae requires careful consideration. To enable a consistent comparative evaluation across all configurations, the elastic modulus *E* in the above equations is replaced by an equivalent elastic modulus *Eeq*, determined using the rule of mixtures based on the volume fractions of the constituent materials:

(5) $$E_{eq}=E_{VW}V_{f,VW}+E_{Ag}V_{f,Ag},$$

where *E_VW_* and *E_Ag_* are the elastic modulus of VeroWhite (1.2 GPa) and Agilus30 (2.0 MPa), respectively [40]; *V_f_*_,*VW*_ and *V_f,Ag_* are the volume fraction of VeroWhite (76%) and Agilus30 (24%), respectively. This yields *E_eq_* ≈ 0.91 GPa. The equivalent Poisson’s ratio is estimated as *υ_eq_* ≈ 0.35 using the same volumetric averaging [41]. It is recognized that this homogenized approach does not fully capture the architectural anisotropy of the overlapping helical composite structure; however, the same *E_eq_* and *υ_eq_* values are applied consistently across all composite structures, ensuring that the comparative rankings of fracture toughness remain valid.

The value of *J* at an instant (*i*) in the loading regime is given by the summation of an elastic component, *J*_el_, and a plastic component, *J*_pl_, as follows:

(6) $$\left\{\begin{array}{c} J_{i}=J_{el(i)}+J_{pl(i)}\\ J_{el(i)}=K_{\left(i\right)}^{2}\left(1-\nu^{2}\right)/E\\ J_{pl(i)}=\left[J_{pl(i-1)}+\left(\frac{\eta_{pl}}{b_{i-1}}\right)\left(\frac{A_{pl(i)}-A_{pl(i-1)}}{B}\right)\right]\left[1-\gamma_{pl}(\frac{a_{\left(i\right)}-a_{\left(i-1\right)}}{b_{\left(i-1\right)}})\right] \end{array}\right.,$$

where $\eta_{pl}$ = 1.9 and $\gamma_{pl}$ = 0.9 are geometry-dependent factors specified in ASTM E1820 for SENB specimens; *b*_(*i*−1)_ is the length of the uncracked ligament; *A_pl_* is the plastic area under the force–displacement curve; *a*_(*i*)_ is the current crack length. The stress intensity factor *K*_(*i*)_ is given as:

(7) $$K_{\left(i\right)}=(\frac{F_{\left(i\right)}L}{BW^{3/2}})f(a_{\left(i\right)}/W),$$

where *f*(*a*_(*i*)_/*W*) is the geometric function given by:

(8) $$f(a_{\left(i\right)}/W)=3{\left(\frac{a_{\left(i\right)}}{W}\right)}^{1/2}\frac{\left[1.99-\left(\frac{a_{\left(i\right)}}{W}\right)\left(1-\frac{a_{\left(i\right)}}{W}\right)(2.15-3.93\frac{a_{\left(i\right)}}{W}+2.7{\left(\frac{a_{\left(i\right)}}{W}\right)}^{2}\right]}{2(1+2\frac{a_{\left(i\right)}}{W}){\left(1-\frac{a_{\left(i\right)}}{W}\right)}^{3/2}},$$

It should be noted that while ASTM E1820 typically evaluates *K*_(*i*)_ at discrete unloading events, all tests in this study were conducted under continuous monotonic loading without intermediate unloading cycles. To apply the ASTM E1820 data reduction procedure, the force–displacement curve was discretized into small increments, and the *J*-integral was evaluated at each increment using the current load, displacement, and crack length. The crack length at each increment was determined from the time-stamped camera images recorded during testing.

The total energy absorption ($E_{total}$) represents the cumulative energy dissipated over the entire bending event. It is calculated as the area under the force–displacement curve from zero displacement to the terminate displacement (*d*):

(9) $$E_{\mathrm{total}}=\int_{0}^{d}F(\delta )d\delta ,$$

where F(δ) and δ represent the instantaneous force and displacement, respectively; *d* is defined as either the displacement at which the specimen completely fractures (i.e., the force drops to zero), or the preset maximum crosshead displacement of 8 mm, whichever occurs first. This integration range encompasses both pre-peak and post-peak deformation, providing a comprehensive measure of the overall energy dissipation capacity of the structure. In addition, to facilitate standardized comparison with future studies that may employ different specimen sizes or masses, the specific energy absorption (SEA), defined as the absorbed energy per unit mass, is also calculated. Since the masses of all tested configurations are essentially equal, the SEA values yield the same comparative ranking as the $E_{total}$. Nevertheless, this normalized metric provides a more universally applicable reference for subsequent research.

The post-peak energy dissipation ratio ($R_{prop}$) is defined as the proportion of absorbed energy after the peak force to the total absorbed energy, which is calculated as:

(10) $$R_{\mathrm{prop}}=\frac{E_{\mathrm{total}}-E_{\mathrm{peak}}}{E_{\mathrm{total}}}\times 100\%,$$

where $E_{peak}$ is the absorbed energy from the onset of loading up to the peak force. A higher ratio indicates a stronger capability of the structure to continuously dissipate energy during the post-peak deformation stage.

## 3. Results Analysis: Mechanical Performance Comparison of Bulk and Bio-Inspired Structures

### 3.1. Characteristics of Force–Displacement Curves

**Figure 3:**
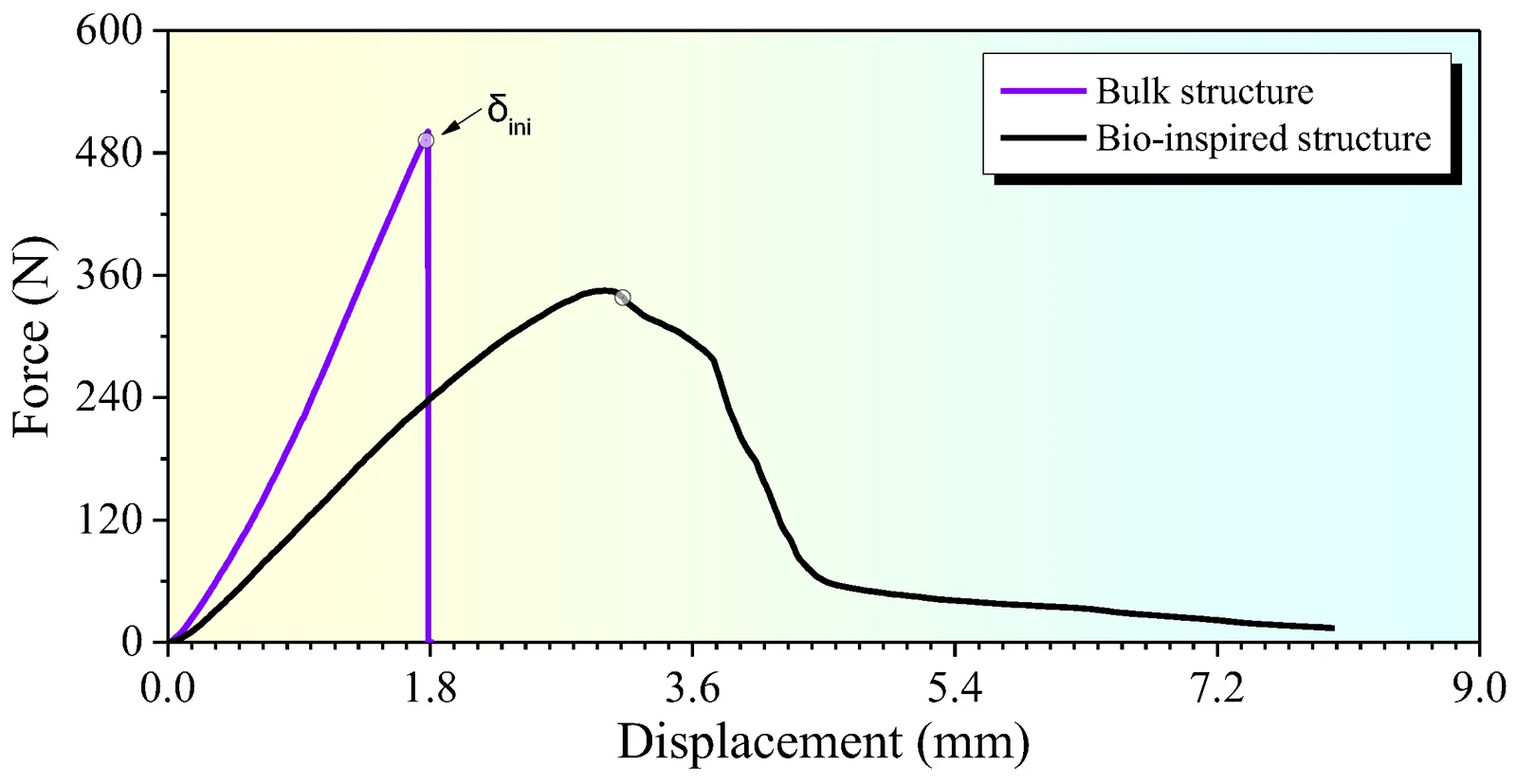
Force–displacement curves of the bulk and fish scale-inspired (*e* = 1.0 and *α* = 19°) structures under quasi-static three-point bending, where the crack initiation displacement ($\delta_{ini}$) is marked.

Figure 3 presents the force–displacement curves of the bulk and fish scale-inspired composite structures (the latter corresponding to the linear helical configuration *e* = 1.0 with a nominal stacking angle *α* = 19°) under the quasi-static three-point bending. The two curves exhibit distinctly different responses, intuitively reflecting the fundamental differences in fracture behavior between the two structures. In detail, the bulk structure exhibits a nearly linear and steep increase during the initial loading stage, demonstrating extremely high flexural stiffness. Upon reaching the peak value of 500.7 ± 18.6 N, the force drops sharply to zero. That is, the bulk structure loses its load-carrying capacity immediately after the peak force, with the entire post-peak process completed within an extremely short displacement range. For the fish scale-inspired structure, its initial slope is significantly lower than that of the bulk (*p* < 0.001), which is attributed to the introduction of the low-modulus soft phase and the presence of soft–stiff interfaces. Its peak force reaches 345.0 ± 11.7 N, which is significantly lower than that of the bulk structure (*p* < 0.001), about a 31.1% decrease. However, beyond the peak value, the curve does not drop abruptly but enters an exceptionally gentle long-term descending regime, with the force decreasing progressively. Therefore, the bulk structure shows an immediate drop to zero after reaching the peak force, whereas the fish scale-inspired structure maintains a prolonged deformation process after the peak.

### 3.2. Comparison of Mechanical Performance Parameters

Table 1 summarizes the specific values of six mechanical indicators for the bulk structure and the fish scale-inspired structure (*e* = 1.0 and *α* = 19°). One-way ANOVA confirmed that the differences between the bulk and bio-inspired structures for all six performance parameters are statistically significant (*p* < 0.001). Regarding flexural modulus, the bulk structure exhibits a high value of 489 ± 17 MPa, while the fish scale-inspired structure shows only 255 ± 8 MPa, a reduction of 47.9%. This phenomenon arises from two factors: the introduction of a soft phase effectively lowers the overall stiffness, and the presence of soft adhesive layers with high shear deformation reduces the effective flexural stiffness by accommodating relative interlayer sliding. In addition, the flexural strength of the bulk structure is 23.47 ± 1.12 MPa compared with 16.17 ± 0.64 MPa for the fish scale-inspired structure—a reduction of 31.1%. For the crack initiation displacement $\delta_{ini}$, it increases from 1.78 ± 0.06 mm for the bulk structure to 3.12 ± 0.11 mm for the fish scale-inspired structure, achieving an improvement of 75.3%. The significant increase in $\delta_{ini}$ for the fish scale-inspired structure indicates that the presence of soft–stiff interfaces delays the onset of macroscopic cracking, thereby improving ductility. In terms of apparent fracture toughness $K_{app}$, it increases from 3.13 ± 0.13 MPa·m^1/2^ for the bulk structure to 5.02 ± 0.17 MPa·m^1/2^ for the fish scale-inspired structure, representing an enhancement of 60.4%. Although the fish scale-inspired structure has a lower peak force, the extensive area under its post-peak softening curve leads to substantially higher $K_{app}$. The total energy absorption increases from 0.40 ± 0.02 J for the bulk structure to 1.03 ± 0.04 J for the fish scale-inspired structure, corresponding to a remarkable enhancement of 157.5%. The SEA shows the same enhancement, increasing from 0.033 ± 0.002 J/g (bulk) to 0.084 ± 0.003 J/g (fish scale-inspired). Notably, the 157.5% increase in total energy absorption is not solely attributable to the 75.3% increase in effective fracture displacement. The fact that the energy absorption increase is more than double the displacement increase indicates a genuine improvement in intrinsic energy dissipation capacity. Under identical quasi-static loading conditions and SENB specimen configurations, the overlapping helical composite structure proposed in this study thus exhibits higher SEA than the BCC (0.032 J/g) and FCC (0.047 J/g) interpenetrating phase composite structure [42] and the bio-inspired helicoidal recursive composite structure (0.056 J/g) [43], achieving significant improvement of about 163%, 79%, and 50%, respectively. Furthermore, the post-peak energy dissipation ratio $R_{prop}$ of bulk structure is only 0.2 ± 0.01%, while the fish scale-inspired structure shows 44.9 ± 1.2%. The extremely low $R_{prop}$ of the bulk structure indicates that there is not stable post-peak deformation stage. Note that for the fish scale-inspired structure, the absolute amount of energy dissipated during the post-peak deformation stage (0.46 J) even exceeds the total energy absorption of bulk structure.

### 3.3. Fracture Paths and Failure Modes

**Figure 4:**
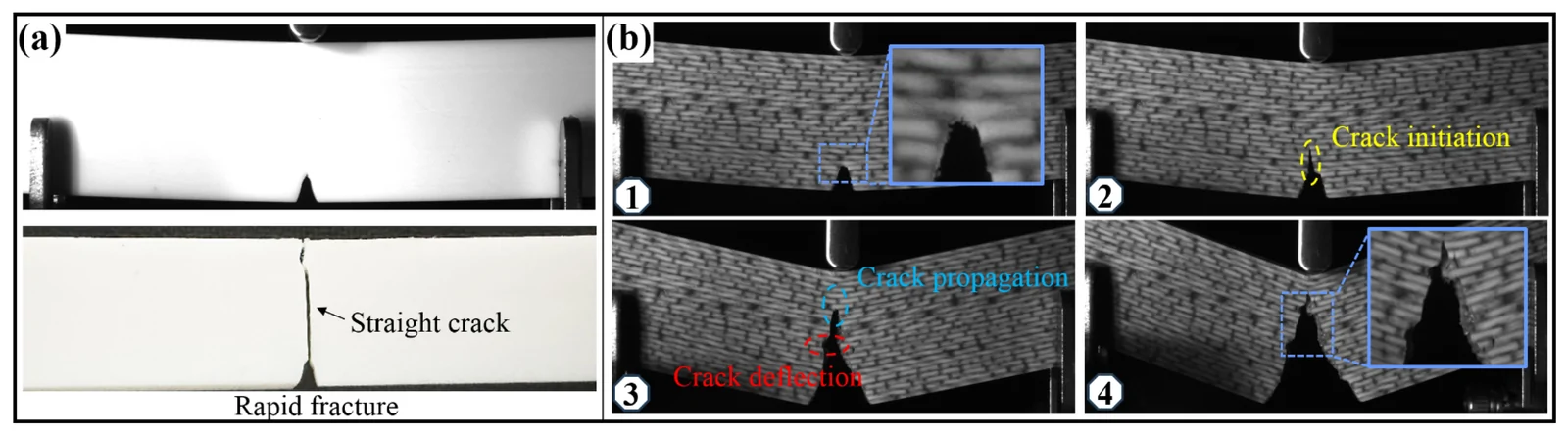
Fracture characteristics of (**a**) bulk structure and (**b**) fish scale-inspired structure (*e* = 1.0 and *α* = 19°). Numbers 1–4 in the subfigures denote the sequential loading stages. The rapid brittle fracture of the bulk structure prevents in situ capture; the fracture morphology is photographed after testing.

The distinct difference in fracture characteristics between the bulk structure and the fish scale-inspired structure (*e* = 1.0 and *α* = 19°) is shown in Figure 4. The bulk structure exhibits a straight and smooth fracture trajectory, propagating vertically from the notch tip through the entire cross-section, with a typical brittle morphology. This straight path indicates that the crack surface area is confined to a single plane, offering extremely limited energy dissipation pathways. This is fully consistent with the vertical post-peak load drop observed in its force–displacement curve. The crack initiation displacement of only 1.78 ± 0.06 mm further confirms this, as the structure undergoes catastrophic fracture within an extremely small deformation range, with virtually no stable propagation stage following crack initiation. By comparison, the fish scale-inspired structure displays a distinctly different crack trajectory. Upon initiation from the notch tip, the crack does not propagate directly upward but undergoes repeated deflections and twists at the soft–stiff interfaces, forming an obvious zigzag path. This tortuous trajectory arises from the overlapping cells and helical layer arrangement, forcing the crack to change direction. This mechanism provides two synergistic toughening effects. First, the crack surface transitions from a single plane to a multi-faceted, multi-directional complex morphology, significantly increasing the total newly created surface area. Second, the total crack propagation length is substantially extended compared with a straight path, dissipating more energy. Collectively, these mechanisms constitute the microscopic physical foundation for the superior $K_{app}$ and $E_{total}$ exhibited by the fish scale-inspired structure. The increase in crack initiation displacement to 3.12 ± 0.11 mm also indicates that the presence of soft–stiff interfaces allows the fish scale-inspired structure to accommodate larger deformation prior to cracking, thereby providing sufficient space for the subsequent activation of crack deflection and toughening mechanisms. The above toughening mechanism analysis is primarily based on macroscopic crack morphology observations. For detailed damage evolution at the microscopic scale, further characterization using techniques such as scanning electron microscopy, micro-computed tomography, or digital image correlation is needed, which will be an important direction for future work. It is worth noting that the observed mechanical performance of the fish scale-inspired structure reflects a typical strength-toughness trade-off. The reductions in flexural modulus and flexural strength are attributed to the introduction of the soft phase, the presence of layered interfaces, and the non-optimal load-bearing orientation of the inclined stiff cells. In contrast, the substantial improvements in apparent fracture toughness and energy absorption mainly arise from the enhanced crack deflection capacity provided by the overlapping helical architecture, which forces the crack to propagate along zigzag paths. This trade-off is characteristic of many biological and bio-inspired composite materials, where the incorporation of compliant interfaces and tortuous crack paths enhances damage tolerance at the expense of stiffness and strength.

## 4. Parametric Studies of Fish Scale-Inspired Composite Structure

### 4.1. Effects of Linear Helical Angle

**Figure 5:**
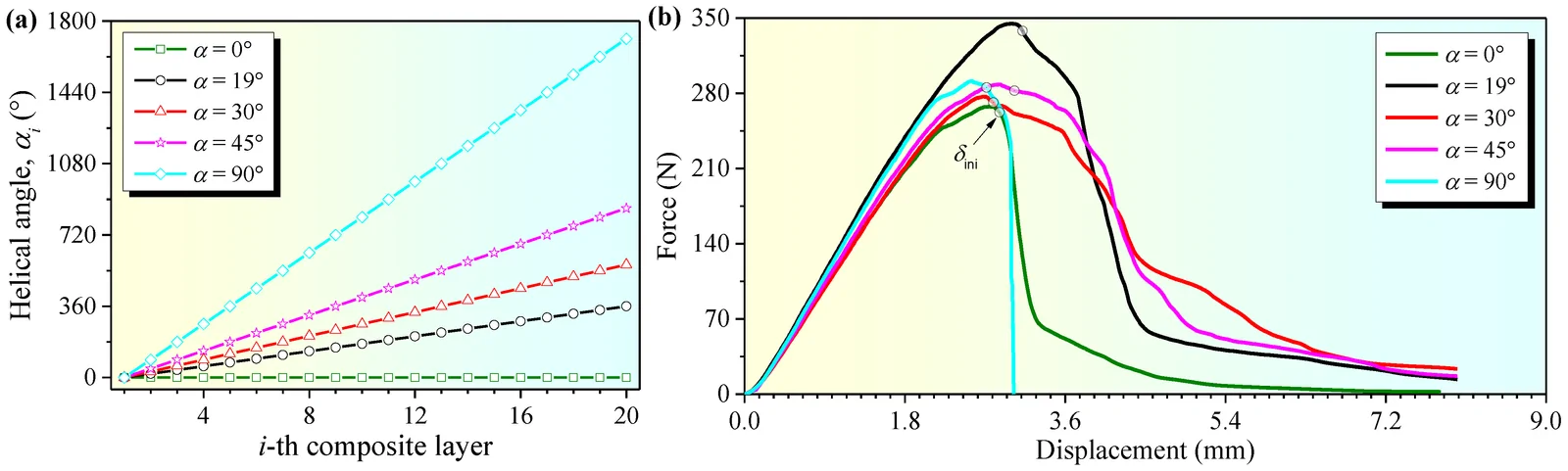
Linear helical angle distribution and mechanical response under quasi-static three-point bending. (**a**) Linear helical angle *α_i_* of the *i*-th composite layer relative to the fixed reference layer. (**b**) Force–displacement curves of the fish scale-inspired structure with different linear helical angles (*α*).

The helical angle is a critical parameter governing the mechanical performance of fish scale-inspired structures. The 19° angle corresponds to the nominal design value derived from the complete helical cycle. In this section, four additional linear helical angles (*α* = 0°, 30°, 45° and 90°) are investigated to examine their effects on the fracture behavior of the fish scale-inspired structure. These angles cover the complete design space from no rotation (0°) to orthogonal crossover (90°), with intermediate values (19°, 30°, 45°) to capture the variation trend of mechanical response. The linear helical angle *α_i_* of the *i*-th composite layer (*i* = 1, 2, 3, …, 20) relative to the fixed reference layer is presented in Figure 5a. Figure 5b shows their force–displacement curves. Overall, the slopes of all curves are comparable during the initial loading stage, indicating that the helical angle has little influence on flexural stiffness. As displacement further increases, however, the curves exhibit pronounced differences near and beyond the peak force. The 0° structure exhibits a rapid force drop after reaching the peak value, with no significant post-peak plateau. The 90° structure also shows a similar response, with the force dropping quickly to a low level after the peak value, indicating extremely limited ductility. In contrast, although the 30° and 45° structures have lower peak force than the 19° structure, they also exhibit a post-peak slowly descending stage, with the 30° structure showing the most pronounced ductile features. Thus, selecting proper helical angles (e.g., 30° and 45°, as well as the baseline 19°) can effectively promote the progressive damage of a fish scale-inspired structure, whereas overly small (0°) or large (90°) helical angles lead to brittle failure.

**Figure 6:**
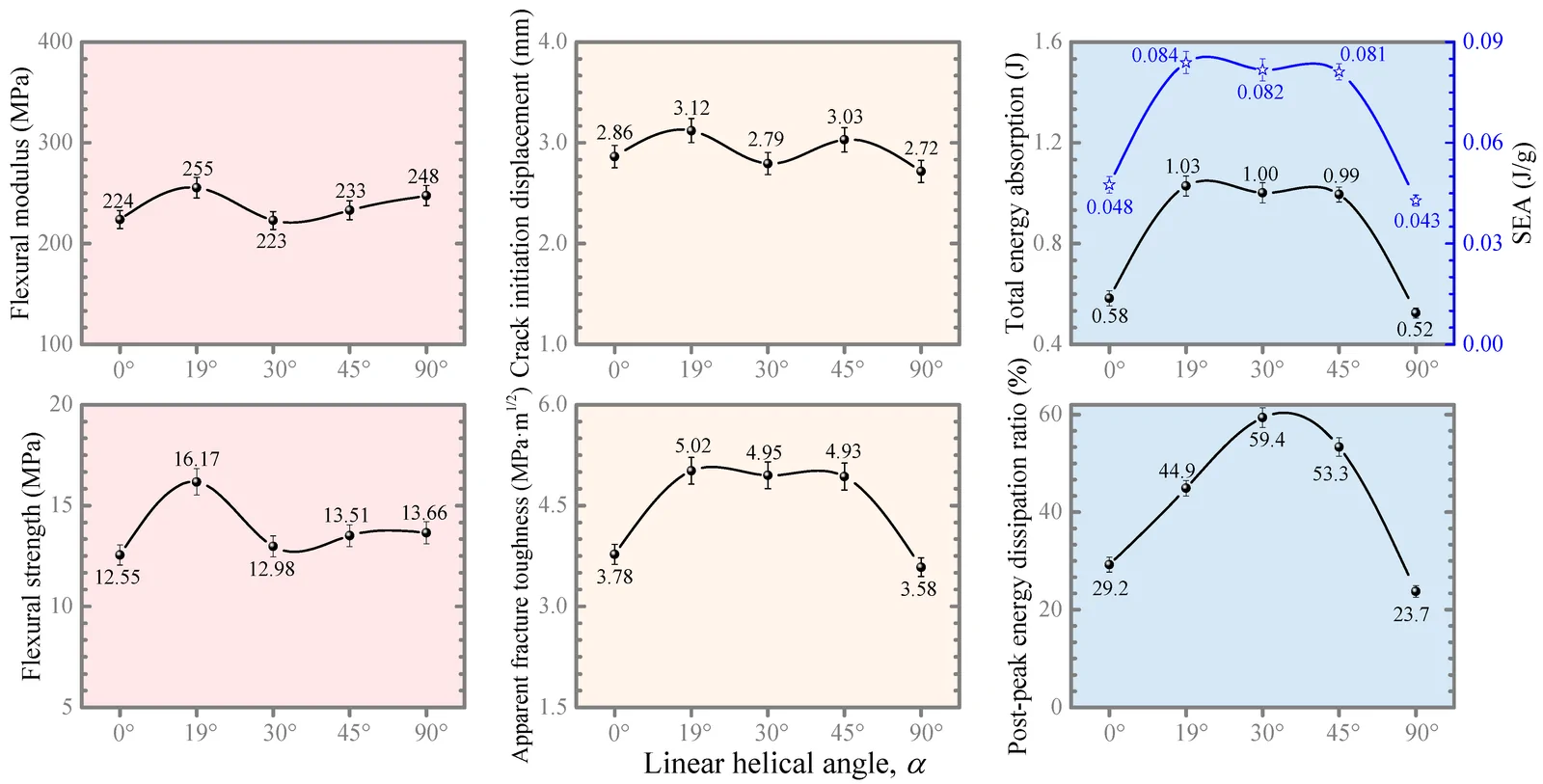
Variation curves of key mechanical performance parameters versus the linear helical angle. Data are presented as mean ± SD.

Figure 6 presents the variation curves of six key mechanical performance parameters versus the linear helical angle. In terms of the flexural modulus, the differences among the selected helical angles are relatively limited, with all values falling within a narrow range of 223–255 MPa. The 19° structure yields the highest flexural modulus, while the 0° and 30° structures yield the lowest. Nevertheless, the overall variation does not exceed 15%. For the flexural strength, the 19° structure again exhibits the maximum value, followed by the 90° structure, while the 0° structure shows the minimum. These results indicate that for both the flexural modulus and strength, an optimal helical angle (19°) exists for the fish scale-inspired structure. Similarly, the crack initiation displacement of the 19° structure is also the largest, followed by the 45° structure. Note that although the 0° and 90° structures also achieve the large crack initiation displacements, which are even larger than that of 30° structure, the poor post-peak responses limit their overall performance. Furthermore, the last three parameters, namely the apparent fracture toughness, total energy absorption (or SEA), and post-peak energy dissipation ratio, exhibit a more definitive and consistent trend, characterized by lower values at both angular extremes and elevated values at intermediate angles. One-way ANOVA confirms that the differences in these three parameters between the extreme angles (0°, 90°) and the intermediate angles (19°, 30°, 45°) are statistically significant (*p* < 0.001). Among the five helical angles, the 19° structure demonstrates the best overall performance across nearly all evaluated parameters, while the 0° and 90° structures exhibit notably poor performance in multiple indicators. Intriguingly, the 30° and 45° structures achieve total energy absorption values comparable to that of the 19° structure, which can be primarily attributed to their extended post-peak deformation stages. This suggests that moderately larger helical angles (30°, 45°) can still provide substantial energy dissipation capacity by prolonging ductile deformation, albeit at the cost of a slight reduction in the flexural modulus and strength. Nevertheless, when considering the comprehensive trade-off among strength, toughness, and ductility, the 19° helical angle remains the most favorable design choice for the fish scale-inspired structure.

**Figure 7:**
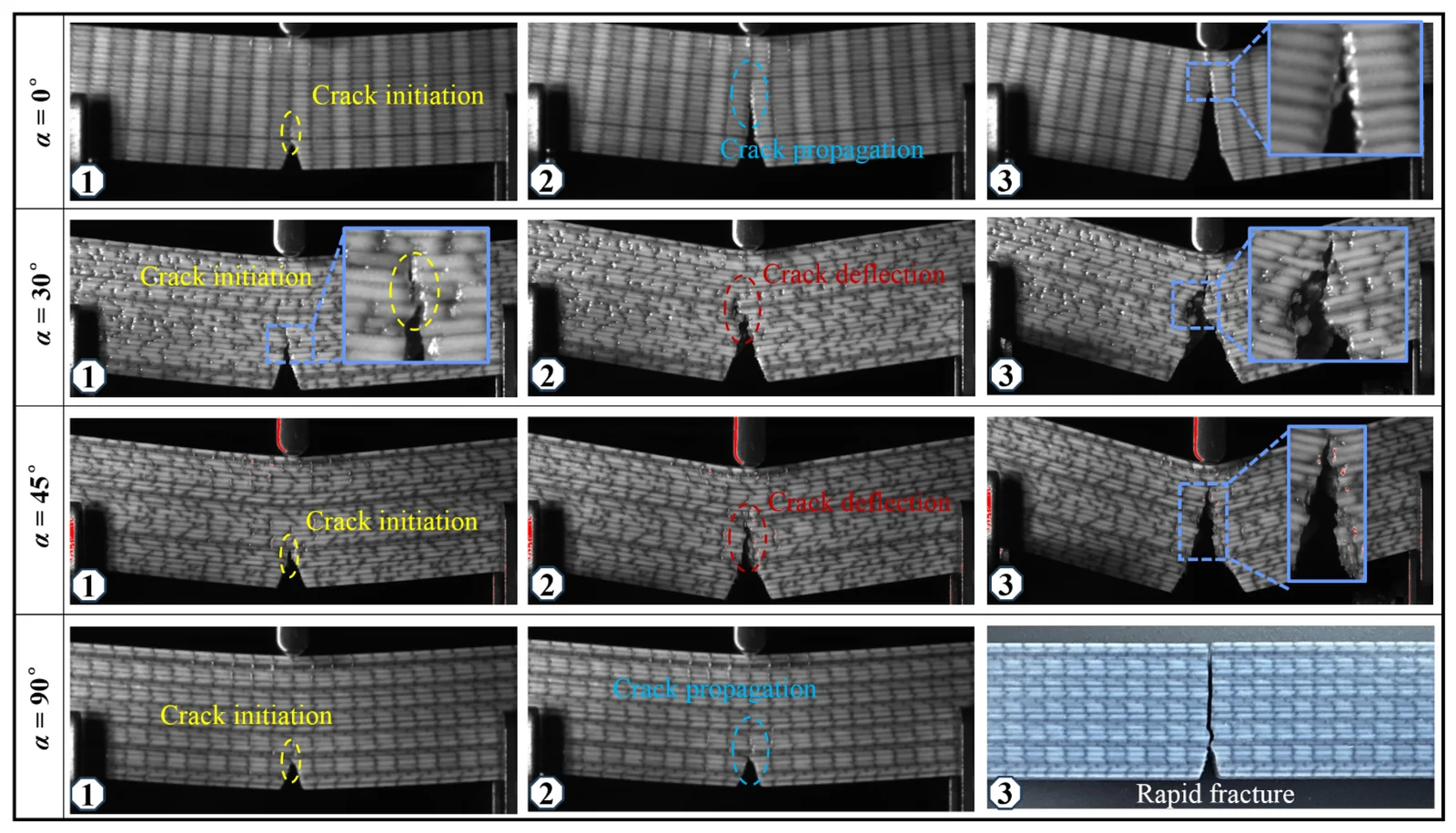
Crack morphologies of the fish scale-inspired structure under different linear helical angles. Numbers 1–3 in the subfigures denote the sequential loading stages. The rapid brittle fracture of the 90° structure prevents in situ capture; the fracture morphology is photographed after testing.

Figure 7 shows the crack morphologies of fish scale-inspired structures under different linear helical angles. It can be seen that their crack propagation paths exhibit significant differences, which directly lead to distinct macroscopic mechanical responses. In detail, for the 0° and 90° structures, the crack propagates approximately straight without any deflection. In contrast, the 19°, 30°, and 45° structures all exhibit crack deflection to varying degrees. Both the 19° and 45° structures display a zigzag propagation path with moderate deflection amplitude, whereas the 30° structure exhibits more obvious deflection and even crack twisting, characterized by large-angle deviations that lead to higher path tortuosity. The disparity in deflection degree directly determines the post-peak mechanical response. The 30° structure, with the most intricate crack path, activates both in-plane deflection and out-of-plane twisting at the interlayer interfaces, thereby forming the most gradual post-peak force decline and exhibiting the most pronounced ductile characteristics. In summary, crack deflection is the core mechanism by which the fish scale-inspired structure achieves toughening. The 0° and 90° structures exhibit brittle fractures because the cracks cannot effectively deflect, restricting energy dissipation to a single crack surface. In contrast, the 19°, 30°, and 45° structures activate multiple energy dissipation mechanisms through crack deflection to different extents, among which the 30° structure achieves the most prominent synergistic effect of deflection and twisting with the best toughening outcome.

### 4.2. Effects of Nonlinear Helical Angle

**Figure 8:**
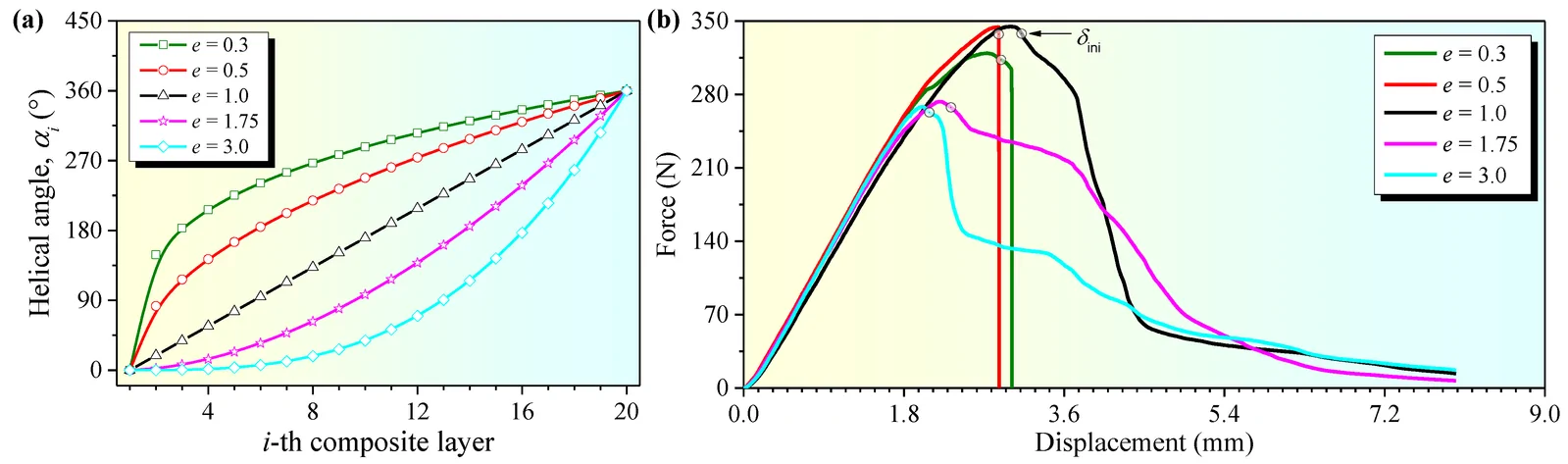
Nonlinear helical angle distribution and mechanical response under quasi-static three-point bending. (**a**) Nonlinear helical angle *α_i_* of the *i*-th composite layer relative to the fixed reference layer. (**b**) Force–displacement curves of the fish scale-inspired structure with different power-law exponents (*e*).

Four power-law exponents (*e* = 0.3, 0.5, 1.75, and 3.0) are investigated to examine their effects on the fracture behavior of the fish scale-inspired structure. The nonlinear helical angle *α_i_* of the *i*-th composite layer (*i* = 1, 2, 3, …, 20) relative to the fixed reference layer is presented in Figure 8a. When *e* < 1, the starting angle at the bottom tensile side is large and decreases with increasing layer number; when *e* > 1, the starting angle is small and increases with layer number. Figure 8b shows the corresponding force–displacement curves. Similar to the linear helical angle, the slopes of all curves are comparable during the initial loading stage, indicating that the initial flexural stiffness is less influenced by the gradient distribution of the helical angle. However, the peak force and post-peak response exhibit characteristics distinctly different from those of the linear helical angle. The *e* = 0.3 and 0.5 structures show a sharp force drop to near zero after reaching the peak, displaying typical brittle fracture characteristics with virtually no post-peak load-bearing capacity. The *e* = 1.75 and 3.0 structures exhibit the lowest peak force but show a certain degree of stepwise gradual decline after the peak.

**Figure 9:**
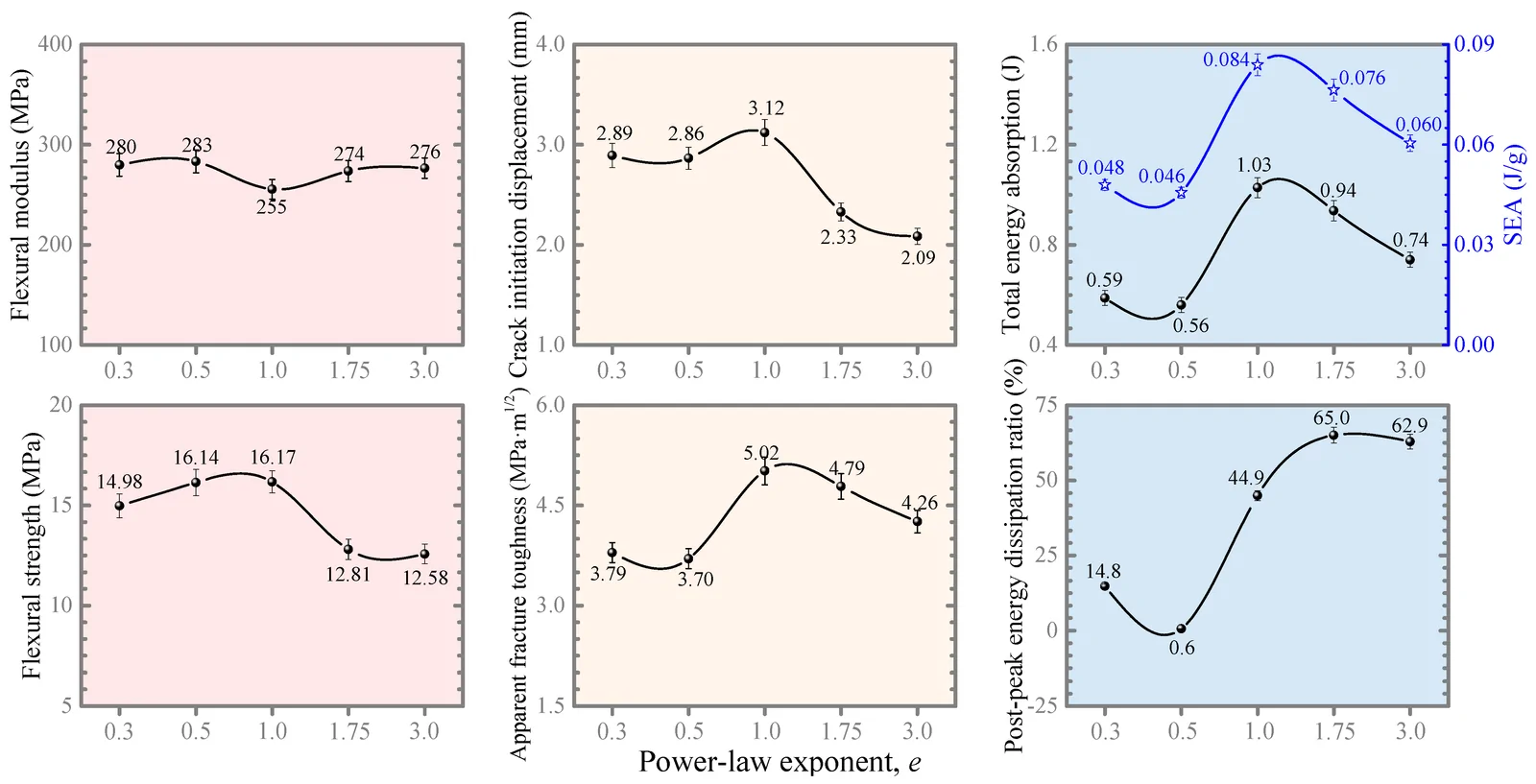
Variation curves of key mechanical performance parameters versus the power-law exponent. Data are presented as mean ± SD.

Figure 9 presents the variation curves of six mechanical performance parameters versus the power-law exponent. For the flexural modulus, the differences among the exponents are relatively limited, with all values falling within the range of 255~283 MPa. The *e* = 0.5 structure achieves the maximum value, while the *e* = 1.0 structure shows the lowest, with an overall variation not exceeding 11%. For the flexural strength, the *e* = 0.5 and 1.0 structures achieve the maximum values, followed by the *e* = 0.3 structure, with the *e* = 1.75 and 3.0 structures being the lowest, exhibiting a trend of high values at intermediate exponents and low values at both ends. For the crack initiation displacement, when *e* ≤ 1, it maintains a relatively high level of 2.86~3.12 mm; when *e* > 1, it decreases to 2.09~2.33 mm. This trend indicates that the small-angle layers at the bottom (*e* > 1) lead to earlier crack initiation on the tensile side. Furthermore, the apparent fracture toughness and total energy absorption (or SEA) exhibit highly consistent change patterns. They remain at low levels when *e* < 1 and increase significantly when *e* ≥ 1, exhibiting a distribution pattern with *e* = 1.0 being optimal. The post-peak energy dissipation ratio exhibits the most obvious variation pattern. It remains at low levels when *e* < 1 and increases substantially when *e* ≥ 1. The *e* = 0.5 structure achieves the minimum value of only 0.1%, indicating that there is almost no dissipative contribution from the post-peak deformation stage. One-way ANOVA confirms that the differences between the *e* < 1 and *e* ≥ 1 groups are statistically significant for apparent fracture toughness, energy absorption, and *R*_prop_ (*p* < 0.001). The *e* = 1.75 structure reaches the maximum value of 65.0%, where energy dissipation is maximally concentrated in the post-peak deformation stage, achieving optimal toughening efficiency.

**Figure 10:**
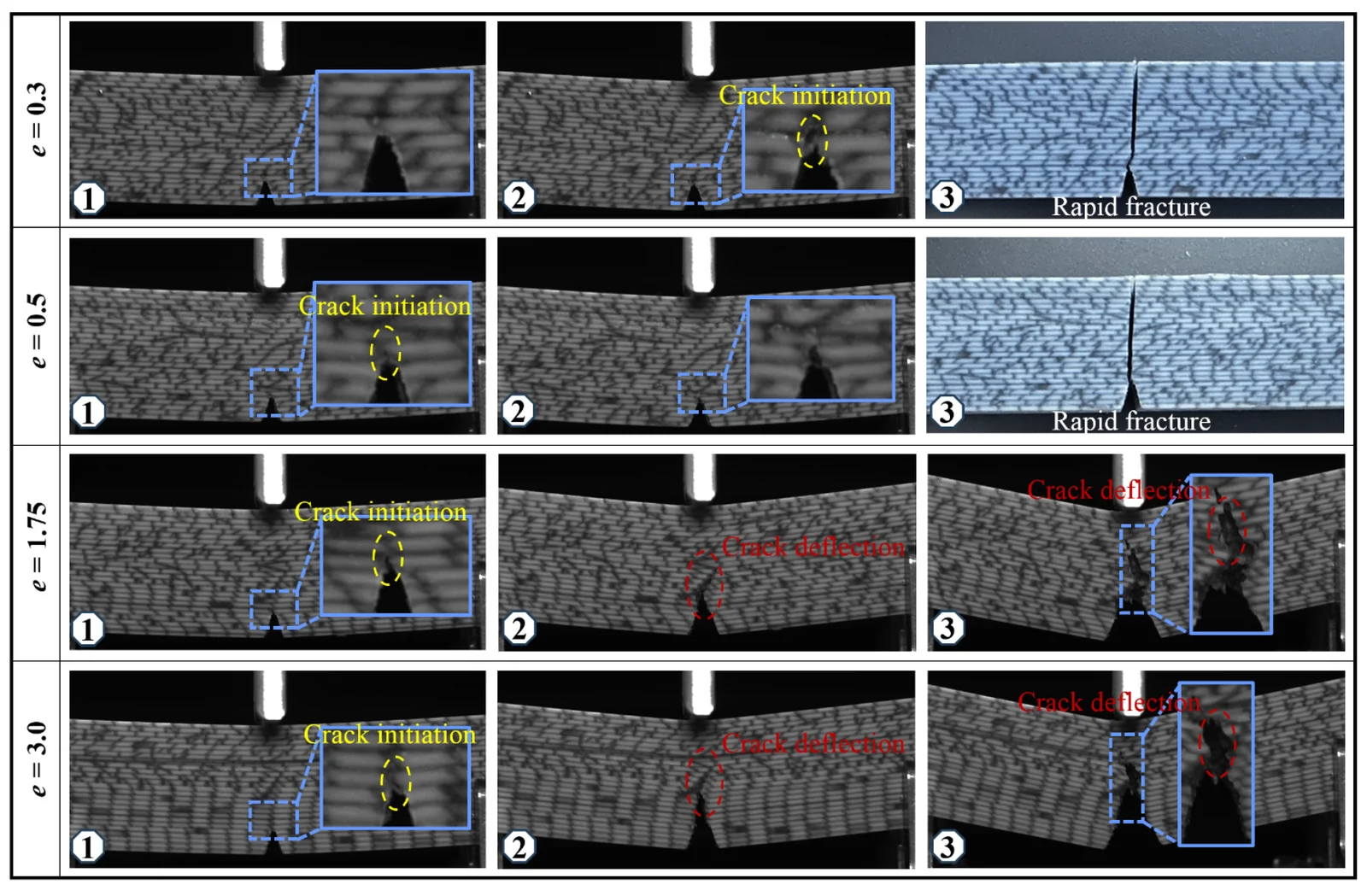
Crack morphologies of the fish scale-inspired structure with different power-law exponents. Numbers 1–3 in the subfigures denote the sequential loading stages. The rapid brittle fracture of *e* = 0.3 and 0.5 structures prevents in situ capture; the fracture morphology is photographed after testing.

Figure 10 presents the crack morphologies of fish scale-inspired structure with different power-law exponents. For the *e* = 0.3 and 0.5 structures, the crack propagates almost vertically through the entire cross-section after initiation from the notch tip, with straight and smooth paths, displaying typical brittle fracture characteristics. These two structures have large helical angles at the bottom tensile side. Once the crack initiates, the lack of small-angle layers as a buffer guide leads to rapid unstable propagation, without forming effective crack deflection. Consequently, energy is dissipated only on a single straight crack surface, without stable post-peak deformation stage. This observation fully corresponds to the vertical post-peak force drop in their force–displacement curves. By comparison, the *e* = 1.75 and 3.0 structures show pronounced deflection characteristics, with the crack first deflecting along the small-angle interfaces at the bottom and then deflecting to the opposite side, exhibiting zigzag propagation. Compared to the *e* = 1.0 structure, these two structures yield larger zigzag patterns and more intricate crack paths. In summary, the design of a nonlinear helical angle plays a significant role in enhancing the mechanical performance of a fish scale-inspired structure, and a moderate nonlinear helical angle is an effective strategy to fully realize the toughening potential of a fish scale-inspired structure. Among all nonlinear helical angle configurations, the *e* = 1.75 structure exhibits the best overall performance. When *e* < 1, large angles at the tensile side lead to late initiation but rapid unstable propagation once cracking occurs, resulting in extremely low *R*_prop_. When *e* > 1, smaller angles at the tensile side allow for earlier initiation, but the progressively increasing angles toward the compressive side promote gradual crack deflection, concentrating energy dissipation in the post-peak deformation stage. The *e* = 1.75 achieves the optimal balance: sacrificing some resistance to crack initiation in exchange for maximizing *R*_prop_ (65.0%) and overall toughening efficiency. However, compared with the linear helical angle configuration (*e* = 1.0), although the apparent fracture toughness is significantly enhanced, the flexural strength and total energy absorption is decreased.

## 5. Conclusions

Inspired by the hierarchical architecture of fish scales, a novel soft–stiff composite structure featuring overlapping helical configuration is proposed. The fracture behavior and toughening mechanisms of the fish scale-inspired composite structure featuring an overlapping helical architecture are systematically investigated. The following key conclusions can be drawn:

(1) The fish scale-inspired structure significantly outperforms the stiff bulk structure in terms of fracture resistance and energy dissipation. While the fish scale-inspired structure exhibits 47.9% and 31.1% lower flexural modulus and flexural strength, respectively, it achieves a 60.4% higher apparent fracture toughness and a remarkable 157.5% increase in total energy absorption.
(2) The toughening mechanism may be attributed to crack deflection, which prolongs the crack initiation displacement by 75.3% and activates a stable post-peak deformation stage, as evidenced by the post-peak energy dissipation ratio increasing from 0.2% for the bulk structure to 44.9% for the fish scale-inspired structure. This transforms the failure mode from catastrophic brittle fracture to progressive damage.
(3) The linear helical angle is a decisive parameter for mechanical performance. An overly small (0°) or large (90°) angle leads to brittle failure with limited energy dissipation. Conversely, intermediate angles (19°, 30°, 45°) effectively promote crack deflection. Among them, the 19° structure offers the best comprehensive balance of strength, toughness, and ductility.
(4) The gradient distribution of the helical angle, governed by the power-law exponent (*e*), significantly alters the fracture behavior. Designs with small angles at the tensile side (*e* > 1) lead to earlier crack initiation yet promote stable post-peak deformation. The *e* = 1.75 configuration maximizes the post-peak energy dissipation ratio (65.0%), concentrating energy dissipation in the crack growth stage and achieving the highest toughening efficiency, though with some sacrifice in flexural strength.
(5) The overlapping helical architecture provides a robust strategy for designing composite structures with high damage tolerance. By rationally tuning the helical angle and its gradient, it is possible to tailor the balance between strength, ductility, and toughness, providing a valuable reference for future engineering applications requiring both protection and flexibility. Other geometric parameters, such as overlap ratio, layer thickness, and helical pitch, warrant systematic investigation in future work to further establish a comprehensive design framework.
(6) Finally, it should be noted that all the experimental results presented in this work were obtained under quasi-static loading conditions (0.5 mm/min). Given the rate-sensitive nature of the polymeric constituent materials, the validity of the observed toughening mechanisms and the optimal design parameters identified herein under dynamic or impact loading requires further investigation. Future work will focus on characterizing the strain-rate-dependent behavior of the overlapping helical structure to fully assess its potential for protective applications.

## Figures and Tables

**Table 1:** Mechanical performance parameters of the bulk and fish scale-inspired (*e* = 1.0 and *α* = 19°) structures. Data are presented as mean ± SD.

Performance Parameters	Symbol (Unit)	Structural Type		Change
		Bulk	Fish Scale-Inspired	
Flexural modulus	$E_{f}$ (N/mm)	489 ± 17	255 ± 8	−47.9%
Flexural strength	$\sigma_{b}$ (MPa)	23.47 ± 1.12	16.17 ± 0.64	−31.1%
Crack initiation displacement	$\delta_{ini}$ (mm)	1.78 ± 0.06	3.12 ± 0.11	+75.3%
Apparent fracture toughness	$K_{app}$ (MPa·m^1/2^)	3.13 ± 0.13	5.02 ± 0.17	+60.4%
Total energy absorption	$E_{total}$ (J)	0.40 ± 0.02	1.03 ± 0.04	+157.5%
Post-peak energy dissipation ratio	$R_{prop}$	0.2 ± 0.01%	44.9 ± 1.2%	/

## Data Availability

The data presented in this study are available on request from the corresponding author.
